# Preventive effects of vitamin D treatment on bleomycin-induced pulmonary fibrosis

**DOI:** 10.1038/srep17638

**Published:** 2015-12-02

**Authors:** Zongmei Zhang, Xiaoting Yu, Xia Fang, Aibin Liang, Zhang Yu, Pan Gu, Yu Zeng, Jian He, Hailong Zhu, Shuai Li, Desheng Fan, Fei Han, Lanjing Zhang, Xianghua Yi

**Affiliations:** 1 Department of Pathology, Tongji Hospital, Tongji University School of Medicine, Shanghai, 200065, China; 2 Department of Pathology, Tumor Hospital of Yunnan Province, The Third Affiliated Hospital of Kunming Medical University, Yunnan, 650000, China; 3 Department of Hematology, Tongji Hospital, Tongji University School of Medicine, Shanghai, 200065, China; 4 Electron Microscopy Core Laboratory, Shanghai Medical College, Fudan University, Shanghai, 200032, China; 5 Department of Pathology, University Medical Center of Princeton at Plainsboro, Plainsboro, NJ, USA; 6 Department of Chemical Biology, Ernest Mario School of Pharmacy; 7 Department of Pathology and Laboratory Medicine, Robert Wood Johnson Medical School and Piscataway, NJ, USA; 8 Cancer Institute of New Jersey, Rutgers University, Piscataway, NJ, USA

## Abstract

Patients with pulmonary fibrosis often have low vitamin D levels, the effects of which are largely unknown. We here report that early vitamin D supplementation significantly reduced the severity of pulmonary fibrosis and inflammatory cell accumulationin in the bleomycin-induced pulmonary fibrosis mouse model on supplementary days 14, 21 and 28 (*P* < 0.001). Vitamin D supplementation also prevented some ultrastructural changes in response to bleomycin administration, including basement membrane thickening, interstitial fibrin deposition and microvilli flattening or disappearance on days 14, 21 and 28, and lamellar body swelling or vacuolation on days 21 and 28. The bleomycin group had rising hydroxyproline level on days 14, 21 and 28, whereas the vitamin D treatment group showed consistently lower hydroxyproline level but still higher than that of the control group (*P* < 0.001). Our immunohistochemistry and densitometry analyses showed less staining for α-smooth muscle actin, a myofibroblast marker, in the vitamin D group compared to the bleomycin group (*P* < 0.001). Thus, vitamin D treatment could prevent bleomycin-induced pulmonary fibrosis by delaying or suppressing ultrastructural changes, as well as attenuating hydroxyproline accumulation and inhibiting myofibroblastic proliferation. These data further our understanding of the roles of vitamin D in pulmonary fibrogenesis and in the treatment of pulmonary fibrosis.

Mortality due to idiopathic pulmonary fibrosis (IPF) has increased over the past two decades to rates of 61.2 deaths per 1,000,000 men and 54.5 deaths per 1,000,000 women per year in the USA^1^. A histological pattern typical of interstitial pneumonia is one of the diagnostic criteria for IPF, including increased interstitial fibrosis (scarring and honeycomb changes) and the presence of myofibroblasts and fibroblasts in fibroblastic foci^1^,^2^,^3^. Myofibroblasts have therefore become a major research focus of IPF^4^. However, understanding of myofibroblasts regulation in IPF remains incomplete.

Vitamin D or its analogs have been associated with fibrosis regulation or found to be useful for treating fibrosis in multiple organs, such as the bone, kidney and liver, but its possible effect on pulmonary fibrosis has not been evaluated. In bones, a 3- to 9-month course of treatment with calcifediol eliminates or significantly decreases bone marrow fibrosis^5^. In addition, intravenous injection of 22-oxacalcitriol significantly improves osteitis fibrosa associated with end-stage renal disease^6^. In kidneys, paricalcitol ameliorates interstitial and perivascular fibrosis in various animal models^7^,^8^. Further studies have revealed that the anti-fibrotic effects observed in kidneys may involve the vitamin D receptor (VDR)/renin-angiotensin pathway^9^, VDR signaling in epithelial-to-mesenchymal transition^10^, VDR/mitochondrial signaling^11^ as well as VDR/TGF-β -SMAD3 pathway^12^. In liver, earlier studies have shown that vitamin D treatment reduces hepatic fibrosis associated with hepatitis C or non-alcoholic fatty liver disease^13^,^14^,^15^, although vitamin D status did not accurately predict the fibrosis stage in a recent hepatitis C genotype 1 cohort^16^.

Vitamin D deficiency has been found in more than half of patients with advanced lung disease^17^,^18^,^19^, suggesting a potential relationship between vitamin D deficiency and advanced lung disease. However, the roles of vitamin D in the pathogenesis and treatment of IPF are largely unknown. We therefore investigated the effects of vitamin D treatment on bleomycin-induced pulmonary fibrosis in mice, with a focus on the roles of vitamin D in its ultrastructural and cellular changes.

## Results

**Figure 1:**
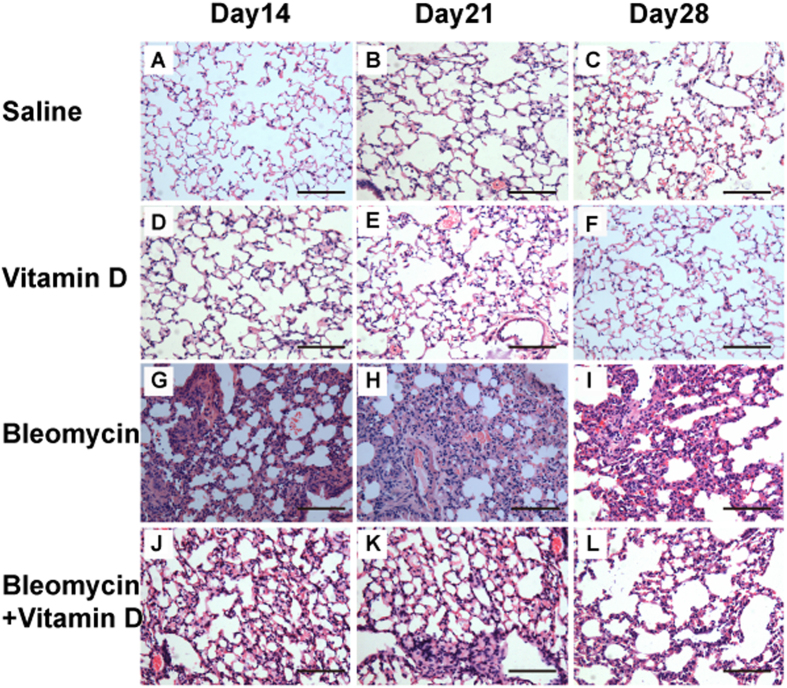
Histological examination showed that vitamin D treatment reduced pulmonary fibrosis induced by bleomycin. The alveoli in the saline group were normal on days 14 (**A**), 21 (**B**) and 28 (**C**). In the bleomycin group, the alveolar septa were thickened, with a significant quantity of inflammatory and interstitial cells present in the pulmonary interstitium on day 14 (**D**); on day 21, fewer inflammatory cells and more interstitial cells were identified in the lung interstitium (**E**). The extent of alveolitis was remarkably reduced, with mainly fibroblast proliferation and collagen deposition, whereas the lung histological architecture was disordered on day 28 (**F**). In the bleomycin+vitamin D group, the extent of alveolitis and pulmonary fibrosis was significantly less than that in the bleomycin group on days 14 (**G**), 21 (**H**) and 28 (**I**). In the vitamin D group, the alveoli were normal on days14 (**J**), 21 (**K**) and 28 (**L**). Bars = 100 μm

**Figure 2:**
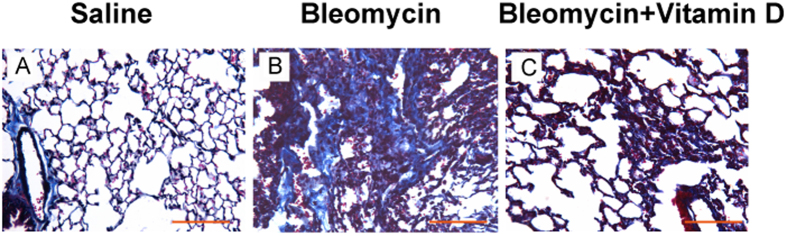
Masson trichrome staining of representative lung tissue on day 21. No significant fibrosis was found in the control group ((**A**), Masson). More collagen deposition/fibrosis in the pulmonary interstitium was found in the bleomycin group ((**B**), Masson). Compared with the bleomycin group, collagen deposition in the vitamin D group was significantly reduced ((**C**), Masson). The experiment was repeated at least three times. Bars = 100 μm

We first observed the histological changes in groups of control, bleomycin, bleomycin + vitamin D, and vitamin D using H&E staining (Fig. 1), Masson trichrome staining (Fig. 2) and Ashcroft’s fibrosis scoring system (Table 1). In the control group, the bronchial epithelium, alveolar epithelium and alveolar wall were all histologically unremarkable (Fig. 1A–C). The Masson trichrome stains highlighted the basement membranes of blood vessels and bronchi which showed no increased interstitial collagen deposition (Fig. 2A). In the bleomycin group, the alveolar septa were progressively thickened on days 14, 21 and 28 (Fig. 1D–F). The number of pulmonary interstitial cells (e.g., fibroblasts) was increased, and the collagen deposition became more and more obvious. Large numbers of inflammatory cells and fibroblasts accumulated in the interstitium led to multifocal myofibroblast clusters, resembling typical interstitial pneumonia. The alveolar structure was partially damaged, and the lung parenchyma became disarranged with focal regions of compensatory emphysema. Notably, alveolitis was most severe on day 14 and subsequently lessened. Pulmonary fibrosis became apparent on day 14 and appeared to peak on day 28 (Figs 1D–F, and 2B). In the bleomycin + vitamin D group, the result showed much less alveolitis and pulmonary fibrosis compared to bleomycin group (Fig. 1G–I). The alveolar septum was slightly thicker compared to the control group but was thinner than that in the bleomycin group (Fig. 2). The numbers of inflammatory cells shown by histologic examination and bronchoaveolar lavage fluid (BALF) analysis were also significantly decreased compared to the bleomycin group (Table 2) (*P* < 0.05). Our fibrosis scoring analysis confirmed that the pulmonary fibrosis observed in bleomycin + vitamin D group was more severe than that in the control group but less severe than that in bleomycin group at all time points (Table 1). In vitamin D group, the toxicity at this dose of vitamin D was analyzed. It showed that the bronchial epithelium, alveolar epithelium and alveolar wall were all histologically unremarkable, similar to those in control group (Fig. 1J–L). Body weights and serum vitamin D concentrations were also measured in all four groups. These results demonstrated that vitamin D is able to alleviate the disease symptoms caused by bleomycin administration. Vitamin D-treated mice exhibited the improved symptoms and signs. Severe adverse effects of vitamin D intoxication (sFig. 1), such as body weight loss, were not observed.

**Figure 3:**
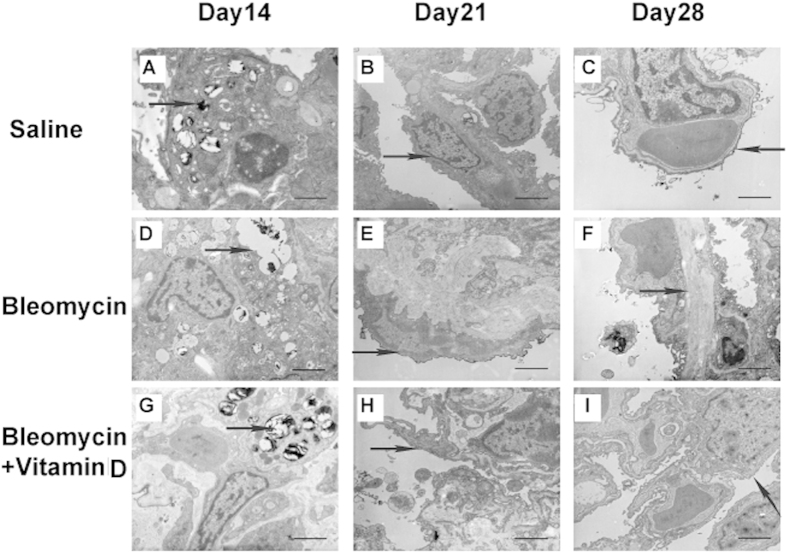
Representative TEM micrographs of the three groups. Saline Group: The alveolar space is patent and clean. Many lamellar bodies with concentric circles or parallel arrangements in the cytoplasm of AT II cells were observed (arrow (**A**)). The structures of type I alveolar epithelial (AT I) cells and AT II cells were integrated. Many short microvilli attached to the free surface of AT II cells were covered with pulmonary surfactant (PS; arrow, (**B**)). There were only thin layers of a basal film between the vascular endothelial cells and type I alveolar epithelial cells (arrow, (**C**)). Bars = 2 μm. Bleomycin Group: Increased numbers of lamellar bodies with obvious evacuation and vacuolation and fusion into macrovesicles were observed (arrows, (**D**)). Part of the microvilli fell off, and the numbers of lysosomes were increased (arrows, (**E**)). Significantly more collagen fibrils were disorganized and deposited in the extracellular matrix. The basement membrane between the vascular endothelial cells and type I alveolar epithelial cells was thickened and filled with collagen fibrils (arrows, (**F**)). Bars = 2 μm. Bleomycin+vitamin D Group: The lamellar bodies showed no evidence of evacuation, vacuolation or cavitation (arrows, (**G**)). The mitochondria were not swollen and the number of lysosomes did not increase (arrows, (**H**)), and the microvilli were well-preserved (**H**). The basement membrane was not significantly thickened on day 28 (arrows, (**I**)). Bars = 2 μm

**Figure 4:**
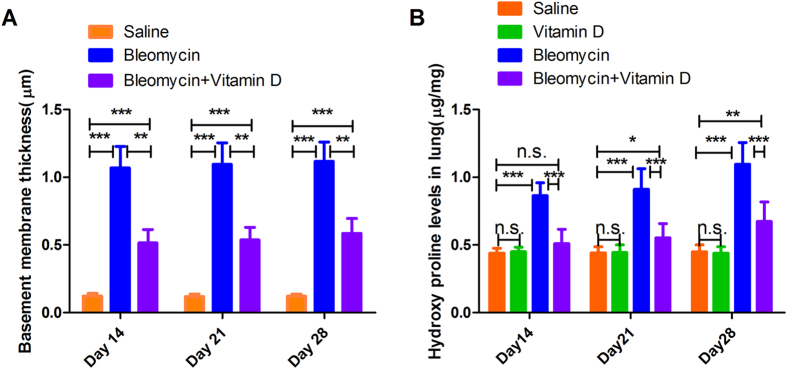
Effects of vitamin D treatment on pulmonary fibrosis. Vitamin D treatment partially reversed the basement membrane thickening induced by bleomycin (**A**). Vitamin D treatment significantly reduced the increase in hydroxyproline content induced by bleomycin, most profoundly on day 14 (**B**). Error bars = standard deviations, N = 6 for each group, n.s. (no significant) *P* > 0.05, *P < 0.05, **P < 0.01, ***P < 0.001.

Bleomycin induces pulmonary fibrosis through characteristic organellar injuries and fibrin accumulation^20^. Thus, we sought to explore the effects of vitamin D treatment on these ultrastructural changes and organellar injuries. Vitamin D treatment indeed suppressed lamellar body swelling or vacuolation, interstitial fibrin deposition and microvilli flattening or disappearance (Table 3, Fig. 3, sFig.3), Unlike interstitial fibrin and microvilli atrophy, the lamellar body swelling or vacuolation was different among the three groups on day 28 but not earlier (sFig. 2), suggesting a time-dependent pattern for the effects of vitamin D. The basement membrane thickness in the bleomycin group was also significantly reduced by vitamin D treatment but remained greater than that in the control group (*P* < 0.001, n = 6 for each group; Fig. 4A).

Lung hydroxyproline levels are a reliable biochemical indicator of collagen content, and we found that on day 14, vitamin D treatment prevented hydroxyproline accumulation in the lung due to bleomycin administration (Fig. 4B). The hydroxyproline contents in the vitamin D+bleomycin were elevated compared to the saline group only on days 21 and 28, but not on day 14. Of note, the hydroxyproline contents in the vitamin D+bleomycin group remained lower than those in the bleomycin group at all time points (Fig. 4B), consistent with our histological and ultrastructural data.

**Figure 5:**
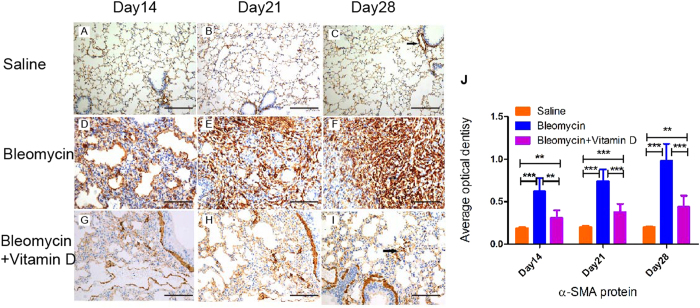
Immunohistochemical staining for α-SMA. Control group on day14 (**A**), day 21 (**B**), and day 28 (**C**). Bleomycin group on day 14 (**D**), day 21 (**E**), and day 28 (**F**). Bleomycin+vitamin D group on day 14 (**G**), day 21 (**H**), and day 28 (**I**). Densitometric analysis (**J**). Error bars = standard deviations, N = 6 for each group, **P < 0.01, ***P < 0.001.

Myofibroblasts are critical for the development of pulmonary fibrosis^21^,^22^, and previous work has demonstrated that vitamin D inhibits the pro-fibrotic effects of lung and skin fibroblasts^23^,^24^. We therefore semi-quantitatively examined whether vitamin D treatment could reduce myofibroblast numbers using α-SMA immuniohistochemical stain, a marker for myofibroblasts, because our qualitative histological analysis showed that this treatment decreased fibroblast numbers (Fig. 5A–C). However, in addition to bronchial and vascular smooth muscles, massive numbers of myofibroblasts expressed α-SMA with a strong stain density in the pulmonary interstitium in the bleomycin group (Fig. 5D–F). The pulmonary interstitium in the vitamin D+bleomycin group had fewer α-SMA-positive cells and a weaker stain density than that of the pulmonary interstitium in the bleomycin group (Fig. 5G–I). Consistently, our densitometric analysis found that vitamin D treatment reduced the increase in α-SMA immunohistochemical staining density induced by bleomycin, but were unable to reduce α-SMA expression back to the levels of the control group (Fig. 5J).

**Figure 6:**
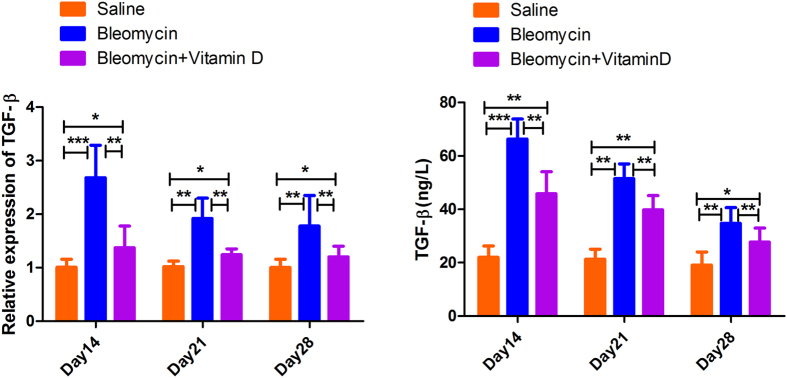
TGF-β was elevated in fibrosis but down-regulated by vitamin D. Real-time PCR analysis of TGF-β in 3 time points of three group (**A**). ELISA analysis of TGF-β protein in BALF (**B**). Error bars = standard deviations, N = 6 for each group, *P < 0.05, **P < 0.01, ***P < 0.001.

As TGF-β1 is considered as the key factor of the pro-fibrogenic factors, we examined its mRNA level in the whole lung and its protein level in BALF. We found that the mRNA and protein levels of TGF-β1 were up-regulated after bleomycin treatment, and the peak was shown on day14. After vitamin D treatment, the TGF-β1 expression levels decreased, which were still higher compared to saline group (*P* < 0.05) (Fig. 6). We also examined the levels of several other classical cytokines (including IFN-γ, IL-4, IL-12, IL-13) and several matrixmetalloproteinases (such as MMP2, MMP9) in this model of lung fibrosis. The levels of those cytokines and MMPs were not significantly changed (*P* > 0.05) (data not shown).

Taken together, our data show that vitamin D treatment inhibited myofibroblast numbers and effectively ameliorated bleomycin-induced pulmonary fibrosis, probably by reducing the basement membrane thickness, preventing organellar injuries, decreasing hydroxyproline accumulation in the lungs and reducing TGF-β expression.

## Discussion

IPF is a specific form of chronic, progressive fibrotic interstitial pneumonia of unknown etiology^1^. Over the last decade, disease definition and diagnostic criteria have evolved significantly, and this has facilitated the design of a number of high-quality clinical trials evaluating novel therapeutic agents for IPF. This massive effort of the medical and industry community has led to the identification of two compounds (pirfenidone^25^ and nintedanib^26^) able to reduce functional decline and disease progression. These promising results notwithstanding, IPF remains a major cause of morbidity and mortality and a large unmet medical need. A real cure for this devastating disease has yet to emerge and will likely consist of a combination of drugs targeting the plethora of pathways potentially involved in disease pathogenesis.

Vitamin D and its analogs play an important role in treating fibrosis in liver, kidneys and bone marrow^6^,^7^,^13^,^27^, which are long-standing, critical targets for fibrosis therapies^28^,^29^. In fact, studies have suggested that vitamin D may be involved in IPF ^17,18,29^. However, the effects of vitamin D on pulmonary fibrosis have not previously been explored, despite *in vitro* evidence supporting its efficacy^24^,^30^,^31^. To the best of our knowledge, we are the first to demonstrate that vitamin D treatment attenuated pulmonary fibrosis induced by bleomycin in mice, suggesting that vitamin D may be potentially used in IPF treatment in human patients. In addition, bleomycin itself is an important chemotherapeutic agent for certain curable tumors, such as Hodgkin’s lymphoma and germ cell tumors^32^. Pulmonary toxicity, including fibrosis, occurs in up to 46% of patients treated with bleomycin, with a mortality rate of 1–3%^32^. Therefore, our *in vivo* data also suggested that vitamin D treatment may help to prevent or ameliorate pulmonary fibrosis in patients treated with bleomycin, a component of chemotherapy regimens. However, confirmatory studies should be conducted.

Our findings may also increase the understanding of the IPF pathogenesis and its potential therapeutic targets which were not well understood before. Bleomycin has been used to produce pulmonary fibrosis in mice since 1974 because of the pathological similarity of bleomycin-induced pulmonary fibrosis and IPF^33^. In the same animal model, the levels of vitamin D, retinyl ester and alpha-tocopherol have been found to be lower than those in a control group^30^. Consistent with this finding, vitamin D deficiency has also been associated with advanced lung diseases and mortality in IPF patients^17^. Our *in vivo* work further supports a role of vitamin D deficiency in the IPF development. The cellular and ultrastructural mechanisms include unchecked myofibroblastic proliferation, injuries to organelles (such as lamellar bodies and microvilli), and increased collagen/fibrin accumulation in the interstitium and in basement membranes. Notably, vitamin D supplementation could not completely normalize the ultrastructural changes and histological injuries due to bleomycin administration. Thus, it is possible that other molecular pathways and ultrastructural changes may also contribute to pulmonary fibrogenesis, such as TNF/TL^34^ and telomerase signaling^35^. Several molecular pathways have been proposed to be involved in the effects of vitamin D on pulmonary fibrosis, with TGF-β1 as a leading candidate. For example, an *in vitro* study showed that vitamin D was able to inhibit the pro-fibrotic effects of TGF-β1 in lung myofibroblasts and epithelial cells^24^, consistent with our findings. Similarly, the TGF-β/Smad family has been shown to be critical for renal and hepatic fibrogenesis^10^,^12^,^36^. Future work will be needed to characterize the involved molecular pathways.

Our study had two major limitations. First, we demonstrated the vitamin D’s protective effects on pulmonary fibrosis in mice. However, the dose-dependence, bioavailability and toxicity are largely unknown, and these topics should be examined in future studies. A detailed pharmacological study would be particularly helpful. Second, despite the ultrastructural mechanisms described here, we did not dissect the molecular targets of the vitamin D-receptor pathway because such an analysis was beyond the scope of the current study. Nonetheless, it would be very intriguing to further characterize the roles of vitamin D-associated molecular pathways, including the TGF-β family^12^,^24^,^36^, hepatocyte growth factor^37^ and others, in pulmonary fibrogenesis.

In conclusion, we showed that vitamin D treatment attenuates pulmonary fibrosis and associated cellular and ultrastructural changes induced by bleomycin in mice. Further effort will be necessary to identify the mechanism(s) involved in vitamin D signaling and explore the possibility of using vitamin D for prevention of side effects in the chemotherapy.

## Materials and Methods

### Animal experiments

The study was approved by the Tongji University Animal Care Committee and conducted according to the Guideline for Animal Experiments. According to *Laboratory Animal Science* (Shi Xinyou Eds, Shi Xinyou Eds, 2000-09-01, ISBN9787801570376 People’s military medical press, China), the vitamin D dosage for mice is 25 ~ 50 times larger than human. We used the 50-fold to calculate the equivalent dosage for mice. Because the human daily vitamin D dose is (0.5 -1.0 μg) per day, suppose average weight of human is 60 Kg, then the calculation formula is (0.5 ~ 1.0 ug)X50/60 kg = (0.42 ~ 0.83)ug/kg. A total of 96 male C57BL/6 mice (20–22 grams, 6–8 weeks old) were randomly assigned into the following 4 groups (24/group): a control group (saline+olive oil; 50 μl of olive oil, Sangon Biotech, Shanghai, China); a bleomycin group (bleomycin+olive oil; 5 mg/kg bleomycin, Nippon Kayaku, Japan; bleomycin diluted in 50 μl of olive oil); a vitamin D+bleomycin group (bleomycin+vitamin D; 0.5 μg/kg 1,25(OH)_2_D_3_, Roche Pharmaceuticals Corp., Shanghai, China;1,25(OH)_2_D_3_ diluted by 50 μl of olive oil); and a vitamin D control group (saline+vitaminD; 1,25(OH)_2_D_3_ diluted by 50 μl of olive oil). A single intratracheal instillation of bleomycin was used to induce pulmonary fibrosis in the bleomycin and vitamin D+bleomycin groups.

From day 2, the mice in vitamin D+bleomycin group and vitamin D control group were treated by gavage once a day with 1,25(OH)_2_D_3_ diluted in olive oil, while the mice in the other two groups were treated with an equal volume of olive oil once a day. The mice were sacrificed and necropsied on days 14, 21 and 28 to investigate the potential time-dependent patterns. The right lower lobes of the lungs were fixed in 10% neutral formaldehyde for routine histological processing. Two small fragments of the left lungs (1 × 1 x 1 mm^3^ each) were immediately fixed in 3% glutaraldehyde and 1% osmium tetroxide for more than 2 hours for analysis by transmission electron microscopy (TEM). The remainder of the tissue was frozen and stored at -80°C.

### Histopathology, TEM, immunohistochemistry and hydroxyproline level analysis

Masson trichrome staining was conducted according to the manufacturer’s protocol (Baso Diagnostics, Inc., China). The H&E and Masson trichrome-stained slides were evaluated by two experienced pathologists in a blinded fashion. The Ashcroft method^38^ was used to assess lung fibrosis, and lung tissue was categorized as follows: grade 0, normal lung; grade 1, minimal fibrous thickening of the alveolar or bronchiolar walls; grade 3, moderate thickening of the walls without obvious damage to lung architecture; grade 5, increased fibrosis with definite damage to the lung structure and the formation of fibrous bands or small fibrous masses; grade 7, severe distortion of the structure and large fibrous areas; grade 8, total fibrous obliteration of fields. For cases in which it was difficult to choose between any two odd-numbered categories, an intervening even-numbered grade was given.

The TEM study was carried out as described previously^39^. Briefly, fixed lung tissue was embedded in epoxy resin 618, sliced (50–60 nm) using an ultrathin microtome LKB-I and then double-stained with uranyl acetate and *Citrus medica* sodium citrate. The tissue samples were then examined and photographed using a PHILIPS CM-120 transmission electron microscope. The formalin-fixed, paraffin-embedded lung tissue samples were subjected to immunohistochemical staining using a rabbit anti-α-smooth muscle actin (α-SMA) antibody (1:100, Dako, Denmark) and to automated immunochemical staining; they were then visualized using the EnVision^TM^ FLEX/HRP method (Dako, Denmark). The cells positive for α-SMA staining were defined according to the presence of cytoplasmic staining compared with appropriate positive and negative controls. For the densitometric analysis of α-SMA immunohistochemistry, 10 randomly selected microscopic fields (×200) were photographed and analyzed using Image-Pro Plus software (Media Cybernetics), using the following formula: mean OD = sum of total integrity OD/total area.

Hydroxyproline levels were measured in frozen lung tissue samples by the acid hydrolysis method using a kit and following the manufacturer’s instructions (Nanjing Jiancheng Bioengineering Institute, China). Each sample was tested 3 times.

### Measurements of serum Vitamin D (calcitriol) levels

Serum levels of vitamin D were determined in mice treated with calcitriol and other three groups on day 28, when the serum concentration of calcitriol already reached a steady state. Mice were sacrificed by cardiac puncture. Serum was isolated from the blood samples after coagulation. Serum samples were stored at −80 °C and then sent to Shanghai Xuhui Center Hospital on dry ice to determine the serum calcitriol levels by radioimmunoassay (RIA).

### Bronchoalveolar lavage and the analysis of bronchoalveolar lavage samples

Alveolar inflammation caused by bleomycin gradually increased and peaked at day 14. Thus, on day 14, the bleomycin-treated mice were anesthetized and tracheostomized, and their airways and lungs were irrigated three times with 500 μl of 1×PBS at 37 °C. Cells were collected by cytospin (3000 rpm, 15 min) and were counted and analyzed by Wright staining.

### Reverse transcription-PCR and Real-time PCR

RNA was isolated by Trizol reagent as per manufacturer’s instructions (Invitrogen, Carlsbad, CA,USA). Reverse transcription-PCR (RT-PCR) assay was performed accordingly with the High capacity cDNA reverse transcription kit (Applied Biosystems, Foster City, CA,USA). The mRNA expression of TGF-β was quantified by using a SYBR green based real-time PCR analysis and the ABI Prism 7900 HT Sequence Detection System (Applied Biosystems, Foster City, CA, USA). Every gene was tested in three replicates and three independent experiments were performed. To quantify the relative changes in gene expression, the 2^−△△CT^ method was used and reactions were normalized to endogenous control gene β-actin expression levels. The primer sequences were as follows: TGF-β forward: 5′TGGAGCCTGGACACACAGTA; TGF-β reverse:5′ GTAGTAGACGATGGGCAGTGG. β-actin forward: 5′ TTCTTTGCAGCTCCTTCG; β-actin reverse:5′ TTCTGACCCATTCCCACC.

### ELISA

The protein levels of TGF-β1 in BALF was measured using sandwich enzyme linked immunosorbent assays (ELISA). Reagents used for the experiments were standard high-quality chemicals from international companies (TGF-β1: R&D systems, Minneapolis, MN, USA). In each group BALF 50 μL was used. The assays were conducted according to the manufacturer’s guidelines. Absorbance at 450 nm was measured with a microplate reader (TECAN, Austria). All samples were run in 3 times.

### Statistical analysis

Continuous data were reported as means ± SD. Comparisons between the groups were performed using one-way analysis of variance (ANOVA) and Fisher’s exact test. Bonferroni and Scheffe tests were used for the *Post Hoc* multiple-comparison ANOVA analyses. The statistical analyses were conducted using Stata software (v11, College Station, TX). A value of *P* < 0.05 was considered statistically significant.

## Additional Information

**How to cite this article**: Zhang, Z. *et al.* Preventive effects of vitamin D treatment on bleomycin-induced pulmonary fibrosis. *Sci. Rep.* **5**, 17638; doi: 10.1038/srep17638 (2015).

## Supplementary Material

Supplementary Information

## Figures and Tables

**Table 1:** Pulmonary fibrosis scores (Ashcroft method) during the course of vitamin D treatment.

Day 14 *			Day 21*			Day 28*			
Scores	Saline n (%)	Bleomycin n (%)	Bleomycin+vitamin D n (%)	Saline n (%)	Bleomycin n (%)	Bleomycin+vitamin Dn (%)	Saline n (%)	Bleomycin n (%)	Bleomycin+vitamin D n (%)
0	4 (66.7)			5 (62.5)			5 (51.4)		
1	2 (33.3)			3 (37.5)			2 (28.6)		
2			2 (40)						
3		2 (33.3)	2 (40)			1 (16.7)			4 (66.7)
4			1 (20)			5 (83.3)			2 (33.3)
5		1 (16.7)			2 (28.6)			1 (14.3)	
6		2 (33.3)			3 (42.9)			1 (14.3)	
7		1 (16.7)			2 (28.6)			5 (71.4)	
Sum	6 (100)	6 (100)	5 (100)	8 (100)	7 (100)	6 (100)	7 (100)	7 (100)	6 (100)

Note: *indicates *P* < 0.001 by Fisher exact test.

**Table 2:** Total cell number and differential cell count in bronchoalveolar lavage fluid of each group on day 14.( 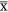 ± s, n = 3).

Group	Total(10^5^/ml)	AM%	PMN%	LYM%	E%
Saline	0.7 ± 0.018	95.1 ± 1.59	1.4 ± 0.6	3.5 ± 1.29	0
Bleomycin	3.34 ± 0.075*	63.2 ± 7.3*	25.1 ± 3.26*	10.5 ± 2.43*	1.2 ± 0.75*
Bleomycin +vitamin D	1.79 ± 0.0392*^, #^	90.7 ± 2.12^#^	4.4 ± 1.29*^, #^	4.6 ± 1.73^#^	0.3 ± 0.13*^, #^

Note: *indicates *P* < 0.05, compared to saline group; #indicates *P* < 0.05, compared with Bleomycin group. AM: alveolar macrophage; PMN: polymorphonuclear;LYM: lymphocyte; E: eosinophils.

**Table 3:** Vitamin D suppresses the ultrastructure changes associated with bleomycin-induced fibrosis.

Day 14	LBSV				IFD				MVF			
	n	Present	None	*P*	n	Present	None	*P*	n	Present	None	*P*
Saline	6	0	6	0.114	6	0	6	0.002	6	0	6	0.004
Bleomycin	5	3	2		5	5	0		5	5	0	
Bleomycin	6	2	4		6	3	3		6	3	3	
+vitamin D												
Day 21												
Saline	8	0	8	0.014	8	0	8	<0.001	8	0	8	0.004
Bleomycin	6	4	2		6	6	0		6	5	1	
Bleomycin	7	4	3		7	3	4		7	3	4	
+vitamin D												
Day 28												
Saline	6	0	6	0.002	6	0	6	0.004	6	0	6	0.021
Bleomycin	6	6	0		6	6	0		6	5	1	
Bleomycin	6	3	3		6	4	2		6	3	3	
+vitamin D												

Note: LBSV, Lamellar body swelling or vacuolation; IFD, Interstitial fibrin deposition; MVF, microvilli flattening or disappearance. Fischer’s exact test was used to calculate the 2-sided P values.
