# Supplementary material for: Preventive effects of vitamin D treatment on bleomycin-induced pulmonary fibrosis
Source: Sci Rep. 2015 Dec 2;5:17638. doi: 10.1038/srep17638 (PMC4667265; doi:10.1038/srep17638)
Supplement: Supplementary Information [file srep17638-s1.pdf]

Preventive effects of vitamin D treatment on bleomycin-induced pulmonary fibrosis

Running title: Vitamin D prevents bleomycin-induced pulmonary fibrosis

Zongmei Zhang[1, 2#], Xiaoting Yu[1#],Xia Fang[3], Aibin Liang[3], Zhang Yu[4], Pan Gu[1], Yu Zeng[1], Jian He[1],Hailong Zhu[1], Shuai Li[1], Desheng Fan[1], Fei Han[1], Lanjing Zhang[5, 6, 7, 8*], Xianghua Yi[1*]

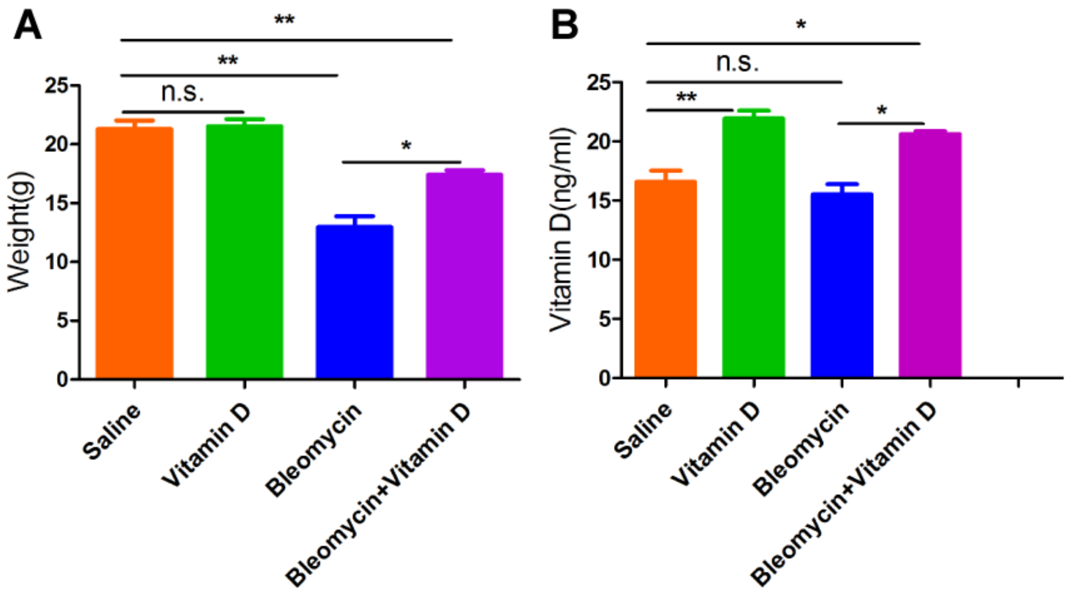

sFig 1 Body weight and serum vitamin D levels in 4 groups on day 28.(n=3) There was no significant body weight difference between the saline and vitamin D treatment groups (A) . Vitamin D treatment partly rescued the weight reduction caused by bleomycin (A) and increased the serum vitamin D levels (B). **P*<0.05, ***P*<0.01.

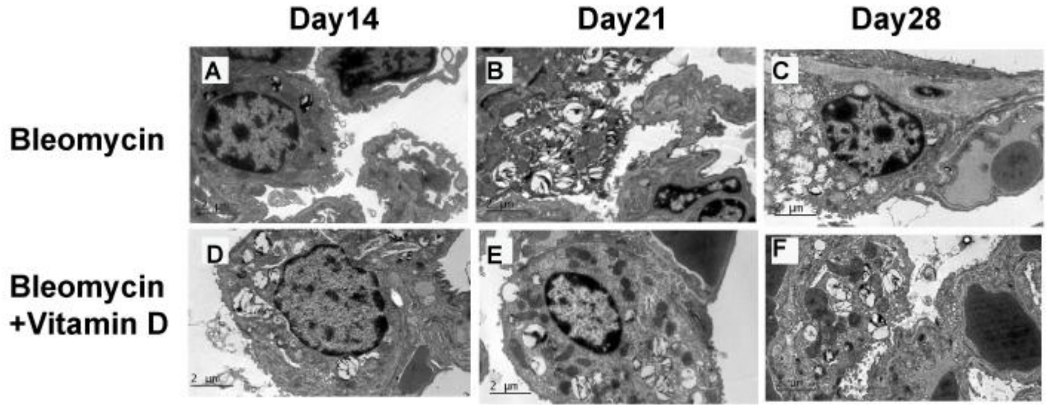

sFig 2 TEM micrographs of the lamellar body in bleomycin group and vitamin D treatment group of three time points. Bleomycin group day14(A), day 21(B), and day 28(C). Bleomycin+Vitamin D group, day 14(D), day 21(E), and day 28(F). The bleomycin+vitamin D group had significant less lamellar body swelling or vacuolation than the bleomycin group on day 28 but not on earlier days. Bars=2 μm

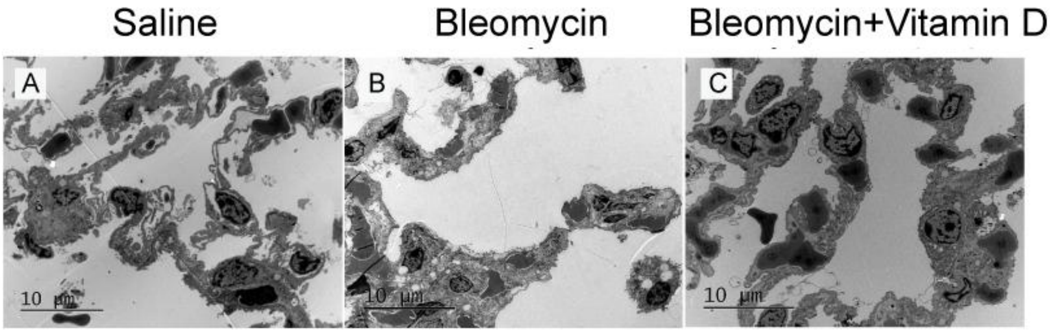

sFig 3 Overview imagine of TEM of three groups at Day 28. Saline group (A), bleomycin group (B) and bleomycin+vitamin D group (C). Bars=10 μm
